# Freely available compound data sets and software tools for chemoinformatics and computational medicinal chemistry applications

**DOI:** 10.12688/f1000research.1-11.v1

**Published:** 2012-08-14

**Authors:** Ye Hu, Jurgen Bajorath

**Affiliations:** 1 Department of Life Science Informatics, Rheinische Friedrich-Wilhelms-Universität, Dahlmannstr, Bonn, D-53113, Germany

## Abstract

We have generated a number of compound data sets and programs for different types of applications in pharmaceutical research. These data sets and programs were originally designed for our research projects and are made publicly available. Without consulting original literature sources, it is difficult to understand specific features of data sets and software tools, basic ideas underlying their design, and applicability domains. Currently, 30 different entries are available for download from our website. In this data article, we provide an overview of the data and tools we make available and designate the areas of research for which they should be useful. For selected data sets and methods/programs, detailed descriptions are given. This article should help interested readers to select data and tools for specific computational investigations.

## Introduction

For compound data mining and the development and evaluation of chemoinformatics methods, public domain databases have become indispensable resources. Currently, major public repositories include PubChem ^1^, BindingDB ^2^, ChEMBL ^3^, and ZINC ^4^. While the former three databases contain compounds and bioactivity data, the latter collects commercially available compounds that are typically not annotated with activity information. Bioactivity data are usually obtained from original literature or patent sources. From these databases, screening data sets (PubChem) and compound activity classes (BindingDB, ChEMBL) can be extracted. Benchmarking of newly developed computational methods typically depends on the availability of such activity classes. Many compounds and measurements are now also shared between these databases. In addition, there are also a number of smaller public and commercial compound databases, which we do not consider here for the purpose of our discussion (with one exception; see below).

Importantly, depending on the scientific questions under investigation, it is often required to design and assemble data sets with specific features. Such data sets, which are usually reported as a part of a publication describing a computational analysis, a new method, or a benchmark investigation, are only infrequently made available to the public. Herein, we describe data sets originating from our laboratory that can be freely obtained. In addition, we also provide information about software tools developed by us that are available via the same website.

## Objectives

The data sets and software tools reported herein have been generated for research activities that essentially fall into four different areas, as reported in Table 1. Area A comprises virtual screening and machine learning applications and is a core area of chemoinformatics. Areas B and C represent molecular selectivity analysis and visualization of structure-activity relationships (SARs), respectively. Furthermore, area D summarizes data mining activities with a focus on structure-activity or -selectivity relationships. Areas B-D are equally relevant for medicinal chemistry (and also chemoinformatics). In addition, especially area B is also relevant for chemical biology. By describing these tools in context, it is hoped that their accessibility to researchers in these areas might be further increased.

**Table 1.** Publicly available data sets and programs. A list of 30 entries providing data sets and/or methods/programs is shown. For each entry, research area indices are assigned as described in the text, i.e. area ‘A’ indicates virtual screening (similarity searching), fingerprint engineering and machine learning; area ‘B’ represents molecular selectivity analysis, area ‘C’ SAR visualization, and area ‘D’ structure-activity or -selectivity relationship-oriented data mining. In addition, publication information is given. For compound data sets, short descriptions are provided. Selected compound data sets are highlighted in red and discussed in the text.

Entry	Year	Area Index	Provided	Data set description
1 ^[8]^	2007	A	Data sets	Nine activity classes (ACs) with increasing structural diversity
2 ^[8]^	2007	A	Data sets	A list of ~1.44 million ZINC compounds used for various virtual screening trials
3 ^[9]^	2007	A	Methods	–
4 ^[10]^	2007	B	Data sets	Four SD files including 26 selectivity sets where compounds are annotated with selectivity values for different tragets
5 ^[11]^	2008	A; B	Data sets	Seven compound selectivity sets containing 267 biogenic amine GPCR antagonists
6 ^[12]^	2008	A; B	Data sets	18 selectivity sets involving targets from four protein families
7 ^[13]^	2008	A	Data sets	25 data sets with compounds of increasing complexity and size
8 ^[14]^	2009	A	Data sets	A set of 242 compounds with hERG inhibitions
9 ^[15]^	2009	A; B	Data sets	A set of 243 ionotropic glutamate ion channel antagonists
10 ^[16]^	2009	C	Data sets; Methods	A sample data set consisting of 51 thrombin inhibitors
11 ^[17]^	2009	A	Data sets	20 ACs assembled from the literature and 15 ACs collected from MDDR
12 ^[18]^	2010	A	Data sets	Eight ACs
13 ^[19]^	2010	B; D	Methods	–
14 ^[20]^	2010	C	Data sets; Methods	A sample data set containing 33 kinase inhibtors
15 ^[21]^	2010	C	Methods	–
16 ^[22]^	2010	C	Data sets; Methods	A sample data set containing 248 Cathepsin S inhibitors
17 ^[23]^	2010	D	Data sets	Two sets of MMPs identified from BindingDB and ChEMBL, respectively
18 ^[24]^	2010	C	Data sets; Methods	A sample data set consisting of 874 factor Xa inhibitors
19 ^[25]^	2010	A	Data sets	17 target-directed scaffold sets where each set contains a minimum of 10 distinct scaffolds and each scaffold represents five compounds
20 ^[26]^	2011	C	Data sets	A list of 10,489 GSK malaria screening hits
21 ^[27]^	2011	D	Data sets	A total of 458 target sets with scaffolds and scaffold hierarchies
22 ^[28]^	2011	C	Data sets	Four data sets containing compounds active against three or four targets
23 ^[29]^	2011	C	Data sets	A set of 881 factor Xa inhibitors
24 ^[30]^	2011	A	Data sets	50 prioritized ACs for similarity search benchmarking
25 ^[31]^	2011	A	Data sets	25 data sets from successful prospective ligand-based virtual screening applications
26 ^[32]^	2011	D	Data sets	A list of 26 conserved scaffolds in activity profile sequences of length four
27 ^[33]^	2011	A	Methods	–
28 ^[34]^	2011	D	Data sets	Two data sets with exclusive K _i_ and IC _50_ measurements
29 ^[35]^	2012	C	Data sets	Four ACs
30 ^[36]^	2012	D	Data sets	Five sets of activity cliffs representing different cliff types

## Materials and methods

Data sets reported herein were mostly, but not exclusively, assembled from BindingDB and ChEMBL on the basis of defined selection criteria (as specified in the original publications). These sets contain compound structures, provided as SMILES ^5^ strings or SD files ^6^, and -whenever appropriate- associated bioactivity information. Some of the older activity classes that are still available from our website have originated from the license-restricted Molecular Drug Data Report (MDDR) ^7^. Therefore, these data sets do not contain compound structures, but only compound identifier information (because a user must obtain a license to access the database). We do no longer license commercial or otherwise restricted databases and will remove corresponding entries from our download section in the near future (to ensure that all compounds are freely available). For the time being, all the information can be accessed. Scripts and programs available from our site generally represent a new computational method or analysis protocol and were implemented in-house in different scripting and programming languages, as specified in the respective entries. Source code is provided. All data sets and programs can be obtained via following URL: http://www.limes.uni-bonn.de/forschung/abteilungen/Bajorath/labwebsite/downloads. The download section is updated several times per year with materials reported in new publications.

## Results and discussion

Table 1 lists all 30 currently available entries including data sets and/or methods/programs. In each case, research area indices are assigned and publication information is provided. Eleven entries are assigned to area A and one, nine, and five entries to area B, C, and D, respectively. In addition, three entries are assigned to areas A and B and one to B and D. Compound data sets originated from 22 different studies and methods from four. In addition, for four other investigations, both methods and data sets are provided.

For compound data sets, short descriptions are provided in Table 1. Furthermore, method/program descriptions are given in Table 2. In a number of instances, data entries contain sets of (filtered) compound activity classes (ACs) to ensure reproducibility of results reported in a specific publication. These sets were often directly taken from BindingDB, ChEMBL, the MDDR, or original literature sources and might not be of above-average interest. Nevertheless, for benchmarking of virtual screening methods, these sets are useful. However, other data sets have been especially designed for novel applications. In the following, selected data sets and methods are described in more detail that might be of particular interest for investigators in the designated (or other) research areas. The indices of these entries are highlighted in Table 1 and Table 2.

**Table 2.** Description of programs and methods. Eight entries with methods/programs are listed. For each entry, a brief description is provided. Selected entries are highlighted in red and discussed in the text.

Entry	Topic	Description
3	Histogram filtering method	A molecular similarity-based method for the identification of active compounds
10	Combinatorial analog graph (CAG)	A methodology that systematically organizes compound analogue series according to substitution sites and identifies combinations of sites that determine SAR discontinuity
13	Target-selectivity patterns of scaffolds	An data mining analysis to identify target-selective scaffolds and their corresponding target-selectivity patterns
14	Multi-target CAG	A methodology for the study of multi-target SARs and identification of substitution sites in analogue series
15	SARANEA	A freely available program to mine structure-activity and selectivity relationship information in compound data sets
16	3D activity landscape	A computational approach to derive 3D activity landscapes for compound data sets
18	Similarity potency tree (SPT)	An intuitive method for visualizing local SARs and prioritizing subsets of compounds of high structural similarity and high SAR information content
27	Scaffold distance function	A quantitative measure of structural distance between molecular scaffolds

### Selected compound data sets

***Entry 1:*** The ACs in this set are designed to have increasing intra-class structural diversity and hence represent test cases of increasing degrees of difficulty for the evaluation of ligand-based virtual screening (LBVS) methods.

***Entry 4:*** In 26 so-called selectivity sets, compounds are organized on the basis of differential potency against pairs of targets as a measure of selectivity. These sets were originally designed to evaluate an extension of standard similarity searching termed selectivity searching. The sets can be used as test cases for any methods that evaluate or predict molecular selectivity, similar to entries 5 and 6. As such, these data sets are relevant for computational chemical biology.

***Entry 5:*** Selectivity sets focusing on the biogenic amine G protein coupled receptor (GPCR) family.

***Entry 6:*** Eighteen sets with further refined selectivity criteria targeting four different protein families.

***Entry 7:*** Twenty-five different sets are provided that contain compounds with increasing topological complexity and molecular size. These compound sets were designed to evaluate molecular complexity effects in similarity searching. They can be utilized to examine the complexity and/or size dependence of a computational method.

***Entry 17:*** Sets of matched molecular pairs (MMPs) are given that were systematically extracted from BindingDB and ChEMBL. An MMP is defined as a pair of compounds that only differ by the exchange of a single fragment (substructure).

***Entry 24:*** On the basis of systematic similarity search profiling of ChEMBL, 50 ACs were selected. These sets represent meaningful test cases for benchmarking of LBVS methods. The ACs were assembled because they were neither too "easy" nor too "difficult" for standard similarity searching using different molecular fingerprints.

***Entry 25:*** This database contains a collection of known active reference compounds, newly identified actives (hits), and screening database information extracted from original literature sources reporting prospective LBVS applications. Only studies were considered that provided sufficiently detailed information to reproduce the search calculations. These studies were identified in a systematic survey of published LBVS applications. The database provides an alternative benchmark system for LBVS. For example, on the basis of these compound sets, it can be determined whether a new methodology is capable of reproducing the results of successful prospective virtual screens using other approaches (i.e., screens that have identified structurally novel and experimentally confirmed hits).

***Entry 30:*** Sets of activity cliffs are provided that belong to five newly introduced structural categories. These cliffs were systematically extracted from ChEMBL (latest release). An activity cliff is defined as a pair of structurally similar or analogous compounds with a large difference in potency. Accordingly, activity cliffs typically represent a rich source of SAR information.

### Limitations of data sets

Entries 1, 4–7, 9, 11, and 12 (assembled until 2010) only contain MDDR compound identifiers, but no structures, due to license restrictions, as commented on above.

## Selected methods and programs

***Entry 10:*** A graphical data structure termed combinatorial analog graph (CAG) is introduced to systematically organize analog series on the basis of substitution patterns and identify subsets of analogs having high in SAR information content.

***Entry 14:*** A further extended and refined CAG implementation for the study of SARs across multiple targets.

***Entry 15:*** SARANEA (a semantic construct of SAR and "Araneae", i.e., the scientific order of spiders) is a collection of different tools for graphical and numerical SAR analysis. It contains the network-like similarity graph (NSG), an SAR network (reminiscent of "spider webs") in which compounds are nodes and edges structural similarity relationships. In addition, nodes are annotated with different levels of SAR information. Several NSG variants have been introduced for different aspects of SAR exploration. The SARANEA tool collection was designed for large-scale SAR data mining and analysis, comparison of global and local SAR features, and the study of structure-selectivity relationships.

***Entry 16:*** A program to calculate and display three-dimensional activity landscapes of compound data sets. An activity landscape is defined as any graphical representation that integrates molecular similarity and activity relationships. A 3D activity landscape can be conceptualized as a 2D projection of a chemical reference space (in which compound dissimilarity increases with inter-compound distance) with an interpolated potency surface added as the third dimension.

***Entry 18:*** The similarity-potency tree (SPT) is a graph representation that organizes compound neighborhoods in large data sets on the basis of structural nearest neighbor relationships and reveals chemically interpretable SAR information. This data structure can be understood as a compound-centric activity landscape view. A basic SPT implementation is also available as a part of SARANEA.

***Entry 27:*** "Scaffold hopping", i.e., the detection of active compounds having different structural frameworks (core structures), is the ultimate goal of LBVS and its primary measure of success. However, the evaluation of the scaffold hopping potential of different LBVS methods is complicated by the fact that scaffold hops can involve similar or different core structures, which is generally not taken into account in the statistical assessment of benchmark investigations. An algorithm is presented that calculates the structural distance between any two scaffolds, regardless of their chemical composition or size. Application of this method makes it possible to quantify the degree of difficulty involved in computational scaffold hopping exercises.

## Conclusions

Herein we have given an overview of specialized compound data sets and methods/programs that have originated from different research projects in our laboratory and that are made freely available to others with interests in chemoinformatics, computational medicinal chemistry, and chemical biology. These tools were presented and described in context. We hope that this report will further alert investigators in our and other scientific fields to available resources for specific computational applications and help to select data sets and tools that are relevant for given research topics. It is also hoped that the introduced methodological concepts will further evolve through wide use by others.
