# Supplementary material for: Freely available compound data sets and software tools for chemoinformatics and computational medicinal chemistry applications
Source: F1000Res. 2012 Aug 14;1:11. [Version 1] doi: 10.12688/f1000research.1-11.v1 (PMC3782340; doi:10.12688/f1000research.1-11.v1)
Supplement: Peer review history

# Peer review: Referee response for version 1

**Published:** 2012-09-04

**Authors:** Michael Gilson

**Affiliations:** 1 Skaggs School of Pharmacy and Pharmaceutical Sciences, The University of California, San Diego, CA, USA

This article takes a valuable step in the direction of improved scientific communication by making compound data sets and software, which have been created by the authors in the course of their research over a number of years, available to the scientific community in machine-readable format.

This step dramatically lower barriers for reproducing, using, and building upon this research and sets an important standard for other authors who write papers about digital collections and software.

Currently, the downloads are provided at the authors’ institutional web-site. Given the possibility that this URL may change, it may be helpful to assign a stable digital object identifier (DOI) to the entire collection of 30 data sets and programs; or potentially to assign a separate DOI to each data set and program, so that they can be referenced and accessed in a more fine-grained manner.

I have read this submission. I believe that I have an appropriate level of expertise to confirm that it is of an acceptable scientific standard.

# Peer review: Referee response for version 1

**Published:** 2012-08-20

**Authors:** Pat Walters

**Affiliations:** 1 Vertex Pharmaceuticals, Cambridge, MA, USA

This work fills a much-needed gap in the molecular modeling and cheminformatics arena. Far too often, authors of computational papers publish work that is difficult, if not impossible, to reproduce.

In many cases, the structures and data used to develop and validate a new method are not included with the paper. When structures are included, they are typically supplied as images. Thus, the only way to reproduce the work is to go through a painstaking, often error prone, process of redrawing structures. If modeling and informatics are to advance, we need to do everything we can to promote reproducibility. We also need to have standard datasets that can be used to readily compare existing methods with new techniques. Datasets like DUD from the Shoichet group and MUV from the Baumann group provide a good start, but we need more. We also need more dialog and critical assessments of these datasets. The datasets provided with this paper will provide important tools for the development, comparison, and evaluation of new computational methods.

In addition to releasing a large number of datasets, the authors have also provided software that allows those reading their papers to critically evaluate their group’s published methods on their own datasets. The release of this software also enables the reader to compare new methods with methods they may already be using. I applaud Hu and Bajorath for their efforts to promote open science and to provide datasets that will hopefully provide the basis for a large body of future work.

I have read this submission. I believe that I have an appropriate level of expertise to confirm that it is of an acceptable scientific standard.
